# Biocompatible Sulphonated PEEK Spheres: Influence of Processing Conditions on Morphology and Swelling Behavior

**DOI:** 10.3390/polym13172920

**Published:** 2021-08-30

**Authors:** Mayelli Dantas de Sá, José William de Lima Souza, Henrique Nunes da Silva, Rodolfo Henrique Nogueira Torres, Michele Dayane Rodrigues Leite, Rossemberg Cardoso Barbosa, Itamara Farias Leite, Cristiane Agra Pimentel, Marcus Vinicius Lia Fook

**Affiliations:** 1 Postgraduate Program in Materials Science and Engineering, Department of Materials Engineering, Federal University of Campina Grande, Campina Grande, PB 58429-900, Brazil; mayellidantas@gmail.com (M.D.d.S.); william.souza@certbio.ufcg.edu.br (J.W.d.L.S.); henrique.nunes.silva.eng@gmail.com (H.N.d.S.); rodolfo_hntorres21@hotmail.com (R.H.N.T.); mdrleite@gmail.com (M.D.R.L.); 2 Department of Materials Engineering, Federal University of Campina Grande, Campina Grande, PB 58429-900, Brazil; rcbvet@gmail.com; 3 Department of Materials Engineering, Federal University of Paraíba, João Pessoa, PB 58051-900, Brazil; itamaraf@gmail.com; 4 Department of Production Engineering, Federal University of Recôncavo da Bahia, Feira de Santana, BA 44380-000, Brazil; pimenca@hotmail.com

**Keywords:** chemical modification, SPEEK, spheres, processing variables

## Abstract

This work aimed to develop and evaluate the influence of processing variables on the morphology and swelling of sulfonated poly(ether ether ketone) (SPEEK) spheres for possible applications as a biomaterial. We used the drip method to obtain spheres with the polymer starting solutions SPEEK-6 (*w*/*v*: 6%) and SPEEK-10 (*w*/*v*: 10%), drip rates (20 and 30 mL/h), and drip heights (5 and 10 cm) in experimental planning. The samples were characterized by Fourier-transform infrared spectroscopy (FTIR), optical microscopy (OM), the absorption capacity of phosphate-buffered saline (PBS) by swelling (%), and statistical analysis of data through Design of Experiments (DOE). The obtained results evidenced that the processing variables influenced the morphology and swelling. Spheres with a bigger concentration of the polymer solution presented a greater degree of sulfonation (DS). We verified that the diameter of the spheres was directly related to the variable height and the sphericity was associated with the speed and viscosity of the solution. Bigger and more pores in a greater amount were observed in the spheres with a greater DS, influencing the behavior of the swelling in PBS. The better variable combinations with a high DS, regular sphericity, a smaller diameter, and greater swelling were the samples S_2_-10-20-5 e S_10_-10-20-5. The cytotoxicity indicated that the best samples obtained in the experimental planning (S_2_-10-20-5 and S_10_-10-20-5) were not toxic. In that regard, the evaluated spheres presented cell viability and swelling capacity, suggesting their possible applications as biomaterials.

## 1. Introduction

Poly(ether ether ketone) (PEEK) is thermoplastic a semicrystalline polymer in the polyaryletherketone (PAEK) class [1,2]. The PEEK monomer is composed of an aromatic ring that is present along the chain and that alternates between an ether bond (–O–) and a ketone group (C=O). Ketone bonds are stronger than ether bonds, which implies material stiffness and restricts the polymer chain rotation. This results in high viscosity and an increase in the vitreous transition temperature (T_g_) and melt crystallization temperature (T_m_) [3].

PEEK has been widely used in the aeronautical industry and started to be used also in the biomedical industry after its biocompatibility certification in 1990 [4]. However, the high glass transition temperature (T_g_) has hindered its processing [5,6,7].

The chemical modification of PEEK with sulfuric acid (H_2_SO_4_) to obtain sulfonated poly(ether ether ketone) (SPEEK) has been an alternative for obtaining bioactive materials due to the easiness to modify the hydrophobic polymer chain through the sulfonation reaction. While the sulfonation process occurs, sulfonic groups (–SO_3_H) are added to the hydroquinone segment indicating the sulfonation degree [8]. The sulfonation degree is a parameter controlled through reaction time, temperature, and sulfonating agent. Consequently, this parameter is fundamental to the SPEEK properties, because the increase of sulfonic groups contributes to higher water absorption, showing that sulfonation is an efficient method to produce membranes and films for biomedical applications [8,9,10].

SPEEK has the potential, when it presents a high degree of sulfonation (DS: >50%), to obtain hydrophilic materials with a high degree of swelling. These characteristics are considered important in the tissue engineering field for the development of potential curatives or controlled release systems [8,11].

The literature still reports few works that focus on the SPEEK processing conditions for its applications as a biomaterial, and researches have been directed to the obtention of membranes. Montero et al. [12] studied the modification of the PEEK structure with sulfuric acid to obtain SPEEK membranes and later diluted it in dimethyl sulfoxide. The technique used to obtain fine films was dip-coating, and the authors also evaluated the influence of different degrees of sulfonation for use in the biomedical field. However, the study only evaluated the time of reaction. Brum et al. [13] evaluated the cytotoxicity behavior in two different sulfonation times, but the results showed that the sulfonation process with a duration of 1.5 h presented limited cytocompatibility when in contact with fibroblasts. Pimentel et al. [7] obtained sulfonated poly(ether ether ketone)/hydroxyapatite by variating the sulfonation reaction time. The study evaluated the cytotoxicity of SPEEK membranes by using L929 cells similar to fibroblasts and obtained s cell viability of 86%, evidencing its applicability as a biomaterial. Sundar et al. [14] obtained SPEEK spheres for drug release, but they did not investigate the cytotoxicity. The study did not evaluate the processing conditions in the sulfonation reaction to reach a high sulfonation degree and obtain the spheres. There are no studies on the processing of SPEEK spheres through a controllable and easy route. Generally, the spherical morphology contributes to a greater contact area and a greater control of the liberation kinetics [9,10]. Additional studies are important for the development of a biomaterial.

SPEEK spheres can be obtained with the drip technique through a system composed of an infusion pump in a bath of cold water. The combination of process parameters such as the drip rate, drip height, and concentration of the polymer solution may affect the material properties. It is then necessary to determine the ideal processing conditions to obtain the spheres. Two characteristics are important for possible applications as biomaterials: form stability and swelling degree, which are directly related to the DS. The greater the DS, the greater the swelling degree and, consequently, the greater the possibility of decreasing the mechanical stability. It is then necessary to have a control of the DS, so that there is a high swelling degree without affecting the mechanical stability of the material. One important aspect in tissue engineering is the liquid absorption capacity of a material, which makes it promising for applications as a biomaterial. Hydrophilic characteristics allow the penetration of biological fluids, easing the incorporation and diffusion of drugs to the medium. Because of that, we found it interesting to evaluate the influence of the processing variables in the diameter and swelling of spheres [15,16].

Therefore, the goal of this study was to obtain and evaluate SPEEK spheres through the drip technique. We used Design of Experiments (DOE) to analyze the influence of processing variables on the morphology, and swelling capacity was studied for possible biomedical applications.

## 2. Materials and Methods

### 2.1. Materials

To obtain SPEEK spheres, the following materials were used: PEEK powder from Victrex—Vicote grade 702 (West Conshohocken, PA, USA) with a molar mass of 100.000 g/mol, a particle size range of 10–50 μm, and a density of 1.32 g/cm^3^, sulfuric acid P.A. 98% (H_2_SO_4_) acquired from Dinâmica (Indaiatuba, SP, Brazil), sodium hydroxide (NaOH) acquired from Neon^®^ (São Paulo, SP, Brazil), and phosphate-buffered saline (PBS, pH = 7.4) acquired from Sigma-Aldrich^®^ (Saint Louis, MO, USA).

### 2.2. Preparation of SPEEK

**Figure 1:**
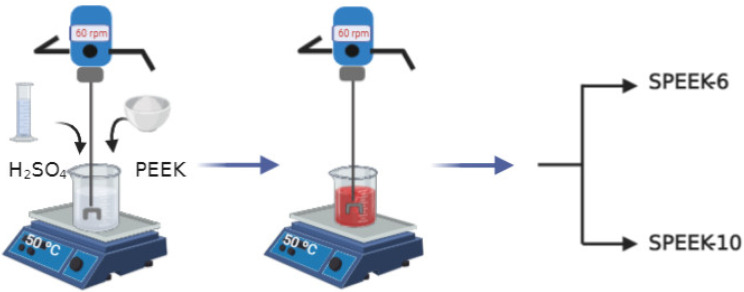
Preparation of a sulfonated poly(ether ether ketone) (SPEEK) polymeric solution.

SPEEK was obtained from the chemical reaction of powder PEEK with sulfuric acid. To obtain the starting solutions, the SPEEK concentrations of PEEK with a *w*/*v* ratio of 6% (SPEEK-6) and PEEK with a *w*/*v* ratio of 10% (SPEEK-10) were used while varying the acid volume. We weighed 2 g of PEEK and used 33 mL of H_2_SO_4_ to obtain SPEEK-6, and for SPEEK-10, we used 2 g of PEEK and 20 mL of H_2_SO_4_. Next, the mixture was heated at 50 ± 1 °C under constant mechanical mixing (60 rpm) for 5 h, as shown in Figure 1. Later, the solutions were used to obtain the spheres. The methodology was based on the works of [12,17,18] with some adjustments.

### 2.3. Preparation of SPEEK Spheres

The method to obtain the spheres starts with the dripping of the SPEEK solution (5 mL in a syringe) in a bath of cold water. The system used was made up of an infusion pump (Pump 11 Pico Plus Elite, Harvard Apparatus, Holliston, MA, USA) with a syringe coupled to a needle with dimensions of 0.7 mm × 25 mm and a magnetic stirrer running at 300 rpm at room temperature (22 ± 1 °C).

The combinations of the starting solutions (SPEEK-6 and SPEEK-10), the drip rates (20 and 30 mL/h), and heights (5 and 10 cm) followed the experimental planning. The solution was dripped in a bath containing 200 mL of distilled water at 9 ± 1 °C under constant magnetic agitation.

**Figure 2:**
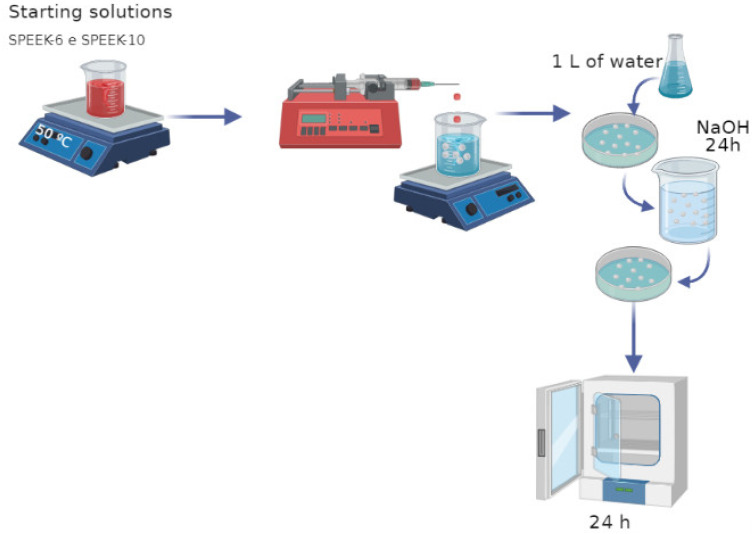
Method of obtaining SPEEK spheres.

After the formation process was over, the spheres were washed with 1 L of distilled water at room temperature (22 ± 1 °C). A 0.5 M sodium hydroxide (NaOH) solution was prepared to neutralize the spheres, which were allowed to stand for 24 h. Next, they were washed again with water and put into a furnace for 24 h, as shown in Figure 2. The samples were encoded according to the starting solution, speed, and drip rate.

### 2.4. Fourier-Transform Infrared Spectroscopy (FTIR)

The FTIR analysis was conducted for the whole spheres at room temperature (23 ± 1 °C) in a Spectrum 400 FT Mid-IR Perkin Elmer spectrometer operating within a wavelength spectrum of 4000–600 cm^–1^. To avoid the production of KBr pellets, an attenuated total reflectance (ATR) device was used. This technique was used to identify the chemical bonds of PEEK and the starting solutions SPEEK-6 and SPEEK-10 and to calculate the DS. Lakshmi et al. [16] evaluated different techniques to calculate the DS, and the results indicated that FTIR may be an efficient, fast and practical technique to determine the DS. The DS was calculated using Equation (1), also described by [19,20,21,22]:

(1) $$\%\mathrm{DS}=\left[1-\frac{\left(\frac{1490}{1471}\right)\;\mathrm{SPEEK}\;\mathrm{peak}\;\mathrm{height}}{\left(\frac{1490}{1471}\right)\;\mathrm{PEEK}\;\mathrm{peak}\;\mathrm{height}}\right]\times 100.$$

The DS was calculated from the correlation of the normalized peak height of SPEEK with the peak height of PEEK. The PEEK sulfonation was confirmed by the division of the group C–C absorption band at 1490 cm^–1^, with the appearance of a new band at 1471 cm^–1^. Therefore, it is necessary to measure the heights of the characteristic bands of both materials [7,12].

### 2.5. Optical Microscopy (OM)

OM was used to observe the morphology of the external surface and to evaluate the transversal cut to analyze the porosity of the spheres. A Hirox Digital Microscope (kh 1300 M, Tokyo, Japan) reflection optical microscope with magnifications of 20×, 60×, and 100× was used. The diameters of the spheres and the average size of pores were measured with ImageJ (Java 1.8.0.112 Version, National Institutes of Health and Laboratory for Optical and Computational Instrumentation, Wisconsin, WI, USA).

### 2.6. Swelling (%)

This experiment aimed to evaluate the behavior of spheres when immersed in PBS. This is a significant experiment, as the absorption capacity may be an important factor for biomaterials with hydrophilic characteristics.

The swelling experiment was performed in triplicate, based on the American Society for Testing and Materials (ASTM) norm D570-98. The samples were submitted to the experiment in PBS with pH 7.4.

Spheres with an amount of 40 ± 2 mg were used as the starting mass of each sample. The times determined for the execution of the experiment were 1, 2, and 7 days. The entire experiment was executed at room temperature (28 ± 1 °C). The samples were withdrawn at the proposed times, and next, the excess of PBS was removed with absorbent paper, followed by weighing in an analytical scale. Equation (2) was used to calculate the degree of swelling degree (SD) measured in percentage:

(2) $$\mathrm{SD}\left(\%\right)=\frac{W_{t}\left(g\right)-W_{0}\;\left(g\right)}{W_{0}\;\left(g\right)}\times 100,$$

where W_t_ and W_0_ represent the weights of swollen and dried-state samples, respectively.

### 2.7. DOE

In this work, a DOE was used to analyze the influence of process parameters on the diameter and swelling of spheres.

We used a 2^3^ factorial planning without center points in duplicate for the statistical analysis. The concentration of the polymer solution, height, and drip rate were analyzed. The experimental DS of the spheres was calculated from the results obtained by FTIR.

The data of the planning variables were processed on the software Minitab 17.1 (State College, PA, USA) and ImageJ (Java 1.8.0.112 Version, National Institute of Health and Laboratory for Optical and Computational Instrumentation, Wisconsin, WI, USA). The experimental 2^3^ factorial planning matrix is shown in Table 1.

The samples and duplicates for the 2^3^ factorial planning are shown in Table 2. The sample name was encoded as follows: the subscript number after the letter “S” is the experiment number, and after that, the next three numbers are the concentration, drip rate, and drip height, respectively. All the experiments in Table 2 were randomly executed.

The response variable of the factorial planning are shown in Table 3. We aimed to evaluate the influence of parameters on the diameters. These results will serve as guidelines to predict the diameter, because the size of the sphere varies according to the application in the biological or pharmaceutical field. The greater swelling of spheres in PBS allowed us to evaluate the hydrophilic behavior and the structural stability of the material in the biological medium. This is an interesting response for applications in dressings and drug carriers, as the liquid absorption by the material may be an important factor in drug release.

### 2.8. Cytotoxicity Study

The cytotoxicity assay was performed on the samples that presented the best results in the experimental planning with a high DS, a smaller diameter, and greater swelling. The cell response was evaluated according to the norm ISO 10993-5: 2013 (Biological evaluation of medical devices—Part 5: Tests for in vitro cytotoxicity). We used the 3-(4,5-Dimethylthiazol-2-yl)-2,5-diphenyltetrazolium bromide (MTT) method in an RPMI 1640 culture medium (Gibco, Roswell Park Memorial Institute, Invitrogen Corporation, Grad Island, EUA).

The L929 fibroblasts (ATCC NCTC clone 929 cell line) were seeded in a 96-well plate at a density of 2 × 10^3^ cells/mL in an RPMI 1640 culture medium. The plate was incubated at 37 ± 1 °C in 5% CO_2_ for 24 h. After that period, the culture medium was aspired, and 200 µL of RPMI 1640 media were added. The plate was again incubated at 37 ± 1 °C under 5% CO_2_ for 24 h.

The culture medium was aspirated from all wells, and next, 100 µL of MTT solutions (1 mg/mL) were added. The plate was incubated again at 37 ± 1 °C under 5% CO_2_ for 3 h.

The supernatant was discarded, and 100 µL of isopropyl alcohol were added per well. Optical density was read on a multi-label plate reader (VictorX3-Perkin Elmer) at 570 nm with 650 nm reference filters. The cell viability was calculated as a percentage of the modified z-score test for outliers detection.

## 3. Results and Discussion

### 3.1. FTIR

**Figure 3:**
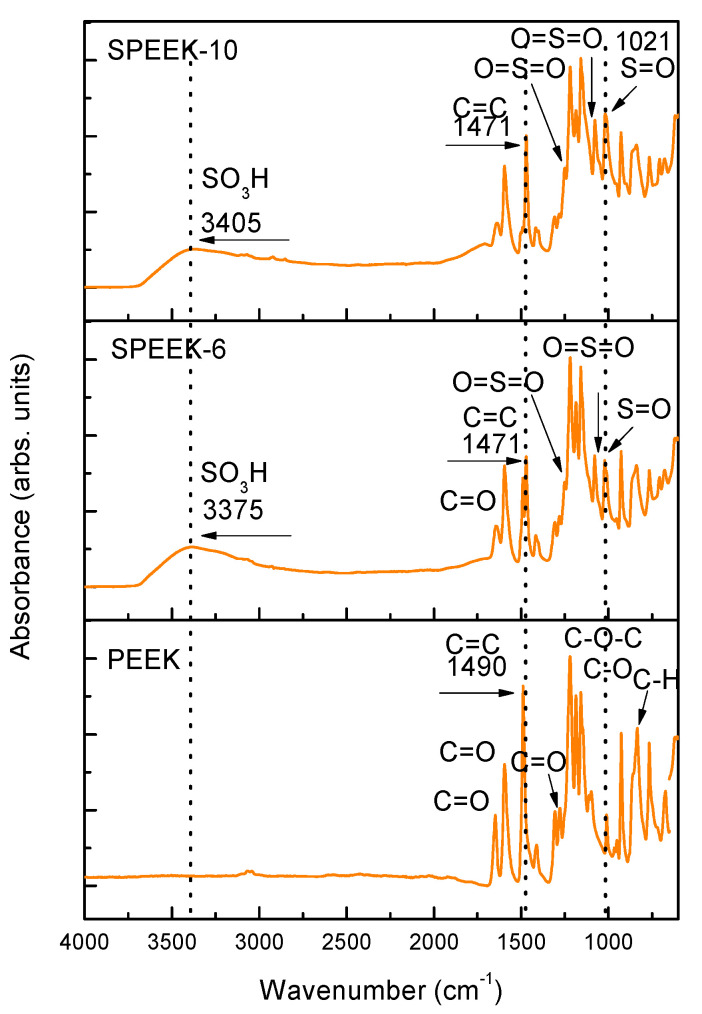
The FTIR spectra of PEEK, PEEK with a *w*/*v* ratio of 6% (SPEEK−6), and PEEK with a *w*/*v* ratio of 10% (SPEEK−10).

The FTIR spectra of PEEK, SPEEK-6, and SPEEK-10 are shown in Figure 3. In the spectrum corresponding to PEEK, the following characteristic bands can be observed: 766, 834, and 925 cm^–1^. They correspond to the angular deformation of the aromatic ring (C–H) bonds. The characteristic bands at 1594 and 1645 cm^–1^ are attributed to the stretching bands of the carbonyl group (C=O) [23].

The sulfonation process occurred, when SO_3_H groups were inserted into the polymeric chain and there was a polymer dissolution in sulfuric acid. After the sulfonation process, we verified the presence of new bands for SPEEK-6 and SPEEK-10 at 1021 cm^–1^, corresponding to the stretching (S=O), and at 3397, 1253, 1077, and 706 cm^–1^, which represent the double bonds between sulfur and oxygen (O=S=O), confirming the sulfonation of the material. Similar results were previously reported in [7,18,24].

The sulfonation was also confirmed by the division of the aromatic absorption band (C=C) at 1490 cm^–1^ and the appearance of a new band at 1471 cm^–1^. These new bands were used to calculate the DS using Equation (1) [7,8,12]. The bands at 3375 and 3405 cm^–1^ correspond to the group (OH), which is bonded to the group SO_3_H. The sulfonic groups are the hydrophilic groups of SPEEK, because they are responsible for increasing the polarity of the polymeric chain and important for the swelling capacity of the material [21].

We noticed that the bands corresponding to the sulfonic groups were more intense as the concentration of the polymeric solution increased. Although there is a correlation between the amount of acid used, which promotes a faster dissolution of the polymer, and the DS, this is not a linear function. For a greater DS, the temperature and duration of the sulfonation reaction may also be controlled [25].

Thus, a greater intensity of this band (SO_3_H) also corresponds to a greater DS, as it was observed in the SPEEK-10 sample. The DSs obtained were 78% for SPEEK-6 and 88% for SPEEK-10, probably due to the amount of acid used [10].

### 3.2. OM

**Figure 4:**
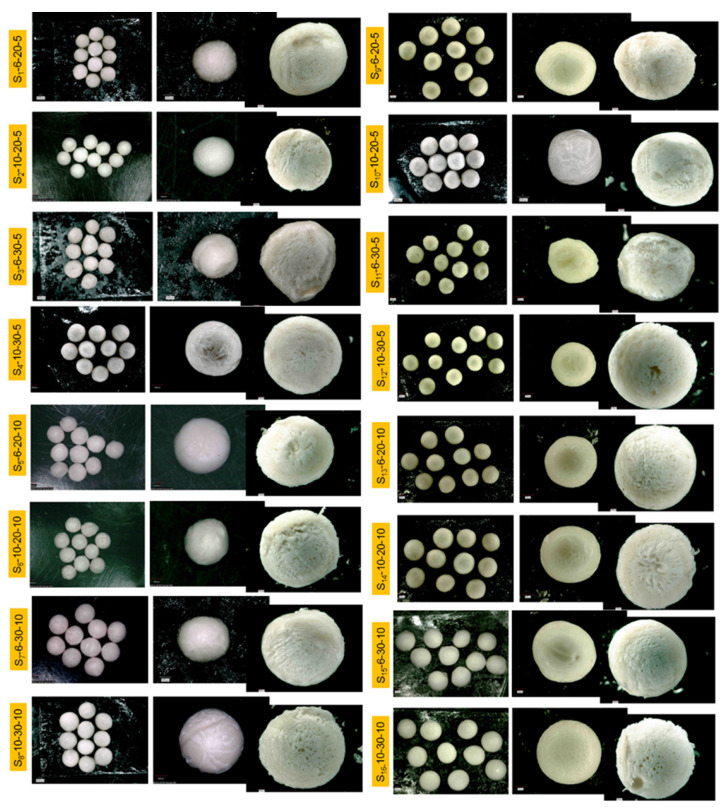
Images obtained by optical microscopy with magnifications of 20×, 60×, and 100× for all spheres of experimental design.

The images of the sphere’s surface with magnifications of 20×, 60×, and 100× are shown in Figure 4. From the images, it can be observed that all the spheres showed rugous surfaces, without agglomerations and a transversal cut with the presence of pores.

The samples S_1_-6-20-5, S_5_-6-20-10, and S_13_-6-20-10 with a DS of 78% showed more regular sphericity when compared to other samples with the same polymer concentration, possibly due to the dripping rate being lower (20 mL/h). On the other hand, the samples S_3_-6-30-5, S_11_-6-30-5, and S_15_-6-30-10 showed a more irregular format, possibly due to the greater drip rate used (30 mL/h). The average diameter of these spheres was 2142.4 ± 236.5 µm.

We can infer that the spheres S_2_-10-20-5, S_10_-10-20-5, S_6_-10-20-10, S_14_-10-20-10, S_4_-10-30-5, S_12_-10-30-5, S_8_-10-30-10, and S_16_-10-30-10 presented a well-defined spherical format with an average diameter of 2300 ± 253.1 µm, probably influenced by the viscosity, as a greater concentration (SPEEK-10) was used. It can also be seen that the drip height was the main variable that influenced the diameters. Spheres with lower diameters were obtained when lower heights were used, and as the drip height grew, the diameter also grew (Figure 4).

From the morphology of the cross-sections of the spheres, it is possible to observe the presence of pores in all the spheres. The amount and size of pores increased for the obtained samples with a high DS (88%). This can be observed in the spheres S_2_-10-20-5, S_4_-10-30-5, S_6_-10-20-10, S_8_-10-30-10, S_10_-10-20-5, S_12_-10-30-5, S_14_-10-20-10, and S_16_-10-30-10. Smaller pores were observed in the spheres S_1_-6-20-5, S_3_-6-30-5, S_5_-6-20-10, S_7_-6-30-10, S_9_-6-20-5, S_11_-6-30-5, and S_15_-6-30-10, as they were obtained with a DS of 78% [26].

We can infer that the spheres S_2_-10-20-5, S_10_-10-20-5, S_6_-10-20-10, S_14_-10-20-10, S_4_-10-30-5, S_12_-10-30-5, S_8_-10-30-10, and S_16_-10-30-10 presented a well-defined spherical format with an average diameter of 2300 ± 253.1 µm, probably influenced by the viscosity, as a greater concentration (SPEEK-10) was used.

**Figure 5:**
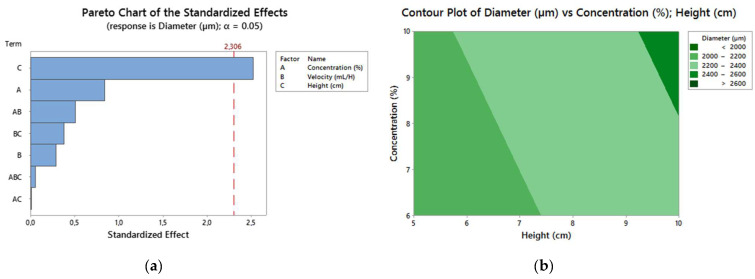
(**a**) Pareto chart; (**b**) contour chart.

It can also be seen that the drip height was the main variable that influenced the diameters. Spheres with lower diameters were obtained, when lower heights were used, and as the drip height grew, the diameter also grew (Figure 5). 

We verified that the pores of all samples presented an average diameter of 57.3 ± 32 µm. The appearance of pores occurs due to the presence of groups SO_3_H that alters the chain conformation and the packing of PEEK, promoting the formation of pores [20]. The presence of pores may cause a greater swelling, since the sulfonation turns the polymer hydrophilic, as proved by FTIR [27].

### 3.3. Swelling (%)

**Figure 6:**
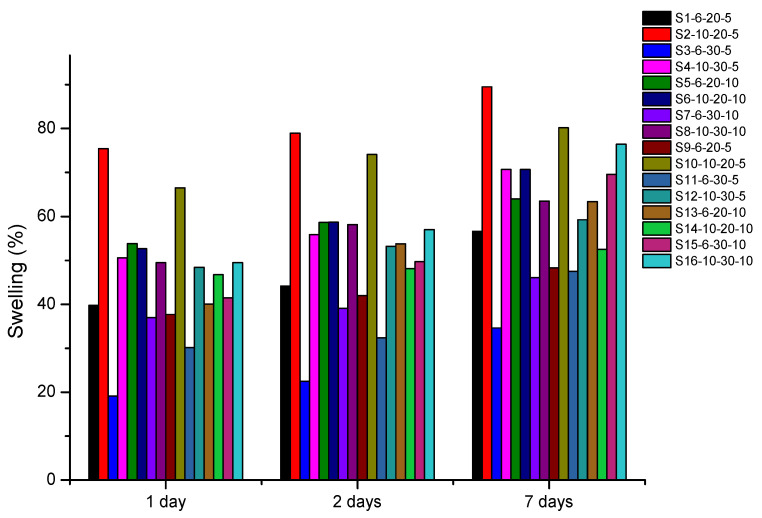
Absorption profile in phosphate-buffered saline (PBS) from all spheres of experimental planning.

The swelling behavior of the spheres in PBS is shown in Figure 6. It is noted that the samples presented the absorption capacity and retention ability of the PBS solution over seven days. This behavior can be attributed to the confirmation of the sulfonation (Figure 3), due to the presence of sulfone groups (SO_3_H) that characterize a strong affinity for polar molecules. Therefore, the polymer began to present a hydrophilic character. These results support the values observed by FTIR [8,11].

Smaller swelling values were observed in the samples S_1_-6-20-5 S_3_-6-30-5, S_7_-6-30-10, S_9_-6-20-5, and S_11_-6-30-5, and the samples S_2_-10-20-5, S_4_-10-30-5, S_8_-10-30-10, S_10_-10-20-5, and S_16_-10-30-10 with a DS of 88% showed greater swelling values. The greatest value was 89% for the sample S_2_-10-20-5.

**Figure 7:**
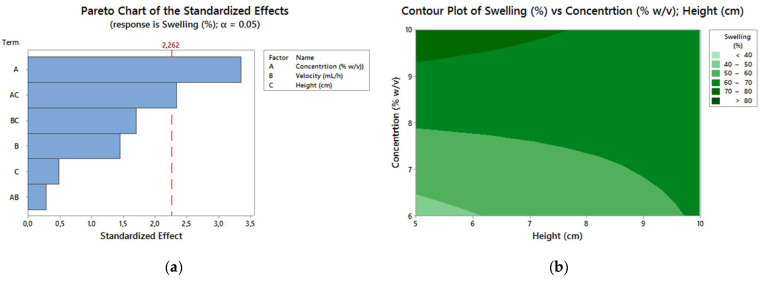
(**a**) Pareto chart; (**b**) contour chart of swelling.

The parameter that had the greatest influence on the swelling was the concentration of the polymeric solution [28,29]. Greater swelling values were observed in the spheres obtained with a bigger concentration (SPEEK-10), as shown in Figure 7. The swelling may be attributed to the porosity of the spheres, since the greater the concentration (SPEEK-10), the greater the DS, presence, and size of pores, resulting in a possible greater PBS absorption capacity.

One of the important aspects of tissue engineering is the liquid absorption capacity of a material, which makes it promising for the biomaterial area. Hydrophilic characteristics allow the penetration of biological fluids, facilitating the incorporation and diffusion of drugs to the medium [30].

### 3.4. DOE Results

The DOE results for the samples are shown in Table 4. This allows us to evaluate the better combination of variables for a high degree of swelling and a smaller diameter.

The response of the optimization analysis performed on Minitab and the multiple response prediction are shown in Table 5 and Table 6, respectively.

Through DOE, it is possible to determine the best parameters in the process of obtention of spheres for possible applications in the biomaterials field. The spheres must show structural stability and high swelling capacity, as these are important characteristics for applications in curatives or drug carrier systems. The diameter of the spheres is also an important response, as it is possible to control and predict their sizes, depending on the application. Consequently, the best combinations of parameters for a greater swelling and a smaller diameter were a high concentration of a polymer solution (SPEEK-10), a low drip speed (20 mL/h), and a low drip height (5 cm). The samples S_2_-10-20-5 and S_10_-10-20-5 presented these combinations. These results support the previous ones, in which the spheres with a greater DS (88%) presented more regular sphericity, a greater pore quantity, a greater diameter, and swelling.

### 3.5. Cytotoxicity Study

**Figure 8:**
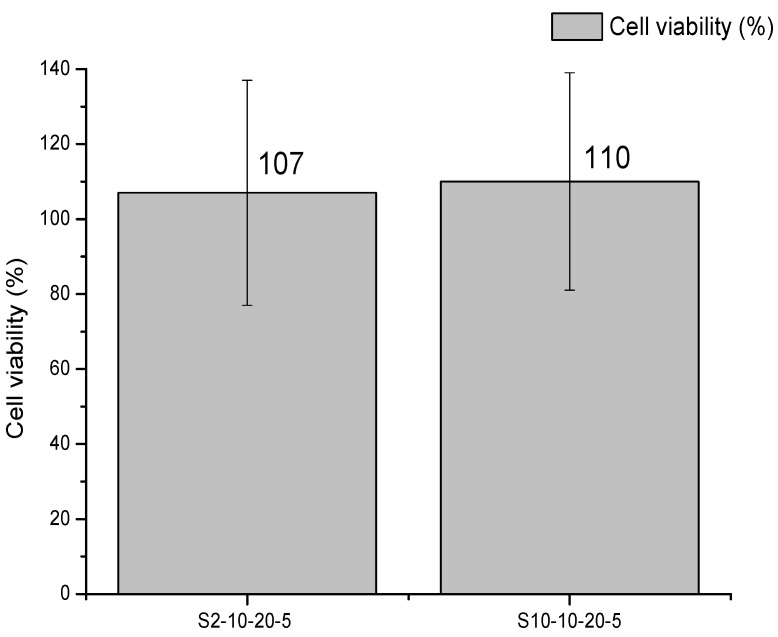
Cell viability of L929 cell lines (when in contact with the S_2_−10−20−5 and S_10_−10−20−5 spheres) by the 3−(4,5−Dimethylthiazol−2−yl)−2,5−diphenyltetrazolium bromide (MTT) method.

Considering the best results of S_2_-10-20-5 and S_10_-10-20-5, the cytotoxicity was evaluated for applications in biomaterials, because toxic substances must not be present. Figure 8 shows the cell viability chart for the best spheres of the experimental planning (S_2_-10-20-5 and S_10_-10-20-5).

It was observed that the metabolic cell activities of spheres were 110% ± 26% for the sphere S_2_-10-20-5 and 107% ± 30% for the sphere S_10_-10-20-5.

When the cell viability of the material is greater than 70%, it is considered non-cytotoxic. The spheres that were evaluated presented cell viability above the cytotoxicity index of 70%, which means that these samples did not present cytotoxic effects when in contact with L929 cell lines, which is in accordance with the literature [7,13].

## 4. Conclusions

The SPEEK spheres were successfully obtained in an infusion pump with the drip technique. The processing variables, such as concentrations, drip rates, and drip heights, influenced the morphology and swelling capacity of the spheres. The DS calculation obtained from the FTIR technique presented a correlation between the concentrations of SPEEK-6 and SPEEK-10. A greater concentration (SPEEK-10) obtained a greater DS (88%). The combination of the concentration (SPEEK-10) and the drip rate (20 mL/h) was determinant for the morphology. We observed a direct relation between the drip height and the diameter of the spheres obtained. The concentration was also significant for the swelling property. A greater concentration (SPEEK-10) resulted in greater swelling.

Thus, the best combinations analyzed through the DOE for obtaining the spheres with a high DS, regular sphericity, greater swelling, and a smaller diameter were the samples S_2_-10-20-5 and S_10_-10-20-5.

The cytotoxicity assay indicated that the best samples obtained in the experimental arrangement (S_2_-10-20-5 and S_10_-10-20-5) were not toxic. The evaluated spheres presented cell viability above the cytotoxicity index. According to the obtained results, we may conclude that the technique used to obtain the SPEEK spheres was effective and the analyzed results suggest potential applications as biomaterials.

## Figures and Tables

**Table 1:** Experimental 2^3^ factorial planning matrix.

Parameters	(−1	+1)
Concentration (%)	6	10
Dripping rate (mL/h)	20	30
Dripping height (cm)	5	10

**Table 2:** Representation of variables and parameters used in triplicates for obtaining the spheres.

Experiment	Sample	Concentration (*w*/*v*; %)	Dripping Rate (mL/h)	Dripping Height (cm)
01	S_1_-6-20-5	6	20	5
02	S_2_-10-20-5	10	20	5
03	S_3_-6-30-5	6	30	5
04	S_4_-10-30-5	10	30	5
05	S_5_-6-20-10	6	20	10
06	S_6_-10-20-10	10	20	10
07	S_7_-6-30-10	6	30	10
08	S_8_-10-30-10	10	30	10
09	S_9_-6-20-5	6	20	5
10	S_10_-10-20-5	10	20	5
11	S_11_-6-30-5	6	30	5
12	S_12_-10-30-5	10	30	5
13	S_13_-6-20-10	6	20	10
14	S_14_-10-20-10	10	20	10
15	S_15_-6-30-10	6	30	10
16	S_16_-10-30-10	10	30	10

**Table 3:** Response variables of the factorial planning.

Response under Analysis	Unit	Characterization
Spheres diameter	µm	ImageJ
Swelling of spheres in PBS	%	Swelling

**Table 4:** Design of experiments (DOE) results for the samples of the experimental planning.

Sample	Diameter of Spheres (µm)	Swelling (%)
	Response I	Response II
S_1_-6-20-5	1846 ± 11	56 ± 9
S_2_-10-20-5	1881 ± 20	89 ± 5
S_3_-6-30-5	2025 ± 14	34 ± 11
S_4_-10-30-5	2280 ± 44	70 ± 7
S_5_-6-20-10	2388 ± 48	63 ± 6
S_6_-10-20-10	2188 ± 37	70 ± 9
S_7_-6-30-10	2279 ± 15	46 ± 7
S_8_-10-30-10	2662 ± 18	63 ± 4
S_9_-6-20-5	2353 ± 23	48 ± 4
S_10_-10-20-5	2378 ± 41	80 ±10
S_11_-6-30-5	2027 ± 13	47 ± 9
S_12_-10-30-5	2087 ± 92	59 ± 3
S_13_-6-20-10	2284 ± 17	63 ± 5
S_14_-10-20-10	2573 ± 66	52 ± 3
S_15_-6-30-10	2441 ± 15	69 ± 9
S_16_-10-30-10	2353 ± 32	76 ± 9

**Table 5:** Response of the optimization analysis made on Minitab.

Solution	Concentration	Rate	Height	Diameter	Swelling	Compound Desirability
1	10	20	5	2133	82	0.82

**Table 6:** Multiple response prediction.

Result	Adjustment	Standard Adjustment Error	95% Confidence Interval
Diameter	2133	(1813; 2453)	(1553; 108.85)
Swelling	82	(67.21; 96.81)	(55.17; 108.85)

## Data Availability

Not applicable.
